# Supplementary figures and images for: Nascent mutant Huntingtin exon 1 chains do not stall on ribosomes during translation but aggregates do recruit machinery involved in ribosome quality control and RNA
Source: PLoS One. 2020 Jul 31;15(7):e0233583. doi: 10.1371/journal.pone.0233583 (PMC7394408; doi:10.1371/journal.pone.0233583)

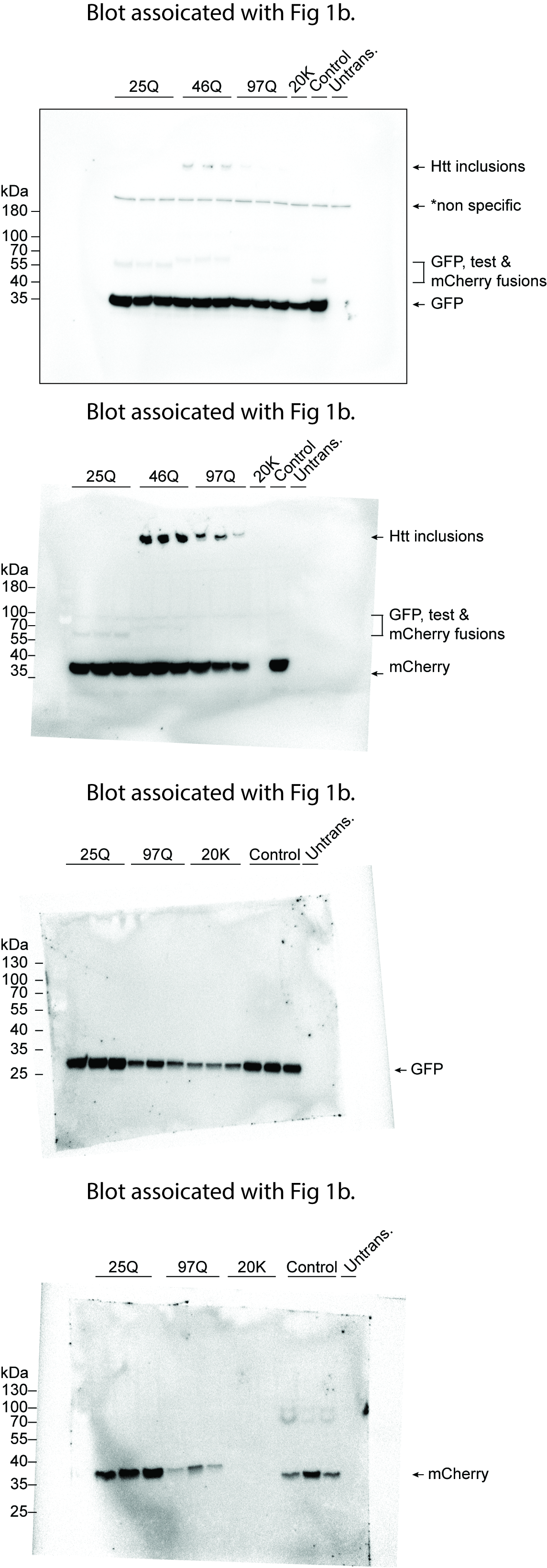

Supplement: S1 Raw images — (TIF) [file pone.0233583.s001.tif]
