# Supplementary material for: Nascent mutant Huntingtin exon 1 chains do not stall on ribosomes during translation but aggregates do recruit machinery involved in ribosome quality control and RNA
Source: PLoS One. 2020 Jul 31;15(7):e0233583. doi: 10.1371/journal.pone.0233583 (PMC7394408; doi:10.1371/journal.pone.0233583)
Supplement: Attachment — Submitted filename: Reviewers Comments.pdf [file pone.0233583.s002.pdf]

Dear Prof Borchelt,

We thank you and the reviewers for your comments and suggestions for improving our manuscript. As indicated below we have made changes to address the reviewers' concerns and used the opportunity to rephrase parts of the manuscript to improve readability and better explain the logic and rationale.

Please see our specific responses below in red.

Additional Editor Comments (if provided):

The authors investigate whether during the translation of mRNA with a long poly Q coding sequence is impaired by ribosome stalling, and whether inclusions that form for expanded poly Q htt fragments show evidence of co-aggregation of proteins known to be involved in ribosome quality control. The paper is somewhat difficult to read due to the extensive use of jargon, but after some effort it is possible to understand the experimental data.
The authors conclude that mutant htt exon 1 mRNAs do not stall ribosomes, and that some ribosome quality control proteins do co-localize with the aggregates.
In my opinion, there are several issues with the paper in its current form that require revision.

1) The htt exon 1 construct used here is not a pure CAG repeat, but instead is a CAGCAA repeat. I would make it clear in the introduction that the design CAGCAA repeat to distinguish nascent polypeptide driven processes from any mRNA driven process that could occur with a pure CAG repeat.

1. We have now included more details in the introduction to make this point clearer.

2) In figure 1B and C, it would seem to me that the western blot data for the P2A T2A vector with 97Q htt is evidence for ribosome stalling. mCherry is missing in the lanes with the 97Q construct. It seems also diminished by 46Q. The fluorescence in C is for the P2A or P2A T2A vectors?

2. We thank the Editor for alerting us to what was indeed a mislabelling of the lanes in the original figure. This error has been corrected. With the correct labelling, the data supports our conclusion for a modest amount of stalling (at best). We have reorganized the panels to make it clearer as to which panel corresponds to which dataset.

3) The technology to distinguish inclusions that may be more mature, or alternatively folded, is interesting, but it really makes the data difficult to follow. In Fig 3, are the examples of fluorescence HBRi or PBRi?

3. We have revised the analysis to provide more transparency to how we have classified the early formed and mature inclusions. Most notably, we have in Fig 2 reanalyzed the data using the specific FlAsH:Cerulean ratios and marked on the figure which values we classify as HBRi and PBRi (which are essentially the opposite ends of the ratio range). Note that these ratios are calibrated for each experiment to a mean and standard error (we have stated the details in the manuscript). For the

other figures we have more clearly marked the FlAsH:Cerulean ratios for the data shown and representative images. All example images in fig 3 and 4 are of cells with inclusions classified as PBRi, which is now stated in the manuscript.

The lines connect data that are from the same cells I believe. In such a case, it may be that paired T-test are more appropriate to compare each category.

4. We have treated these groups as connected with a repeated measure one way ANOVA, which is akin to doing a paired T-test (but for multiple groups at once). This is stated in the figure legend.

What statistics were used to analyze aggregation levels?

5. In the original manuscript we did not assess differences in the data statistically. We have now added a statistical test treating each bin of Cerulean fluorescence (ie expression level proxy) as independent groups versus the treatment groups (halo tag levels) by a two way ANOVA. For this we compared treatments of halotag expressions binned to no expression, low, medium and high expression.

What is the significance of Cerulean fluorescence levels? Is it inclusion size? Is there any meaning if only the brightest inclusions are diminished?

6. Cerulean levels are a measure of huntingtin abundance in cells and in the case where we are not labelling with FlAsH (which we did not do for flow cytometry) this is a suitable proxy. We have modified the figure legend to state this.

Is there any meaning to differences in co-aggregation for a give protein between HBRi and PBRi?

7. We appreciate that we had not clearly articulated the rationale or conclusions of the experiments in Fig 3 and 4. In essence, we sought to examine at what stage of inclusion formation the co-recruited proteins became enriched - and whether the expression of these could affect the inclusion assembly process. Of the proteins that co-aggregated, there was not a strong preference for any of them in early-formed versus mature inclusions but there are some subtle trends. We have now more clearly explained these findings in the results section and the biological implications in the discussion.

4) Similar question as above for Figure 4.

8. Please see our response as above in point 7.

5) What are the different color symbols Fig. 5A and B? Are these individual inclusions? If both the HBRi and PBRi are really just a continuum of the same type of aggregate, what is the value in distinguishing them and are they really different enough to merit separate analyses in Figs. 2-4? Data in Fig 5 suggest that these are structurally similar inclusions.

9. The different colours represent timecourses of individual inclusions. We have now revised this figure (and the legend and results section) to articulate that more clearly. In line with our revised approach to assigning HBRi and PBRi status, we have

revised the plot to show FlAsH:Cerulean ratios as well as the threshold we used to classify the inclusions into the HBRi and PBRi categories. The data suggests that indeed the inclusions appear to not be different in structural composition.

Reviewers' comments:

Reviewer's Responses to Questions

Comments to the Author

Reviewer #1: In this study, Ormsby et al. showed that that expression of wild-type or mutant Httex1 does not stall on the ribosome during translation using a reporter assay, but proteins (Ltn1 and Rack1) involved in ribosome quality control were co-recruited into the inclusions. Additional data showed Hspb1, Sgta, and Snu13 proteins were enriched in the mutant Httx1 inclusions and could suppress the aggregation. These findings provide additional tools to understand the role of the ribosomal quality control machinery in Huntingtin's diseases. Overall, this study is well designed and this is an interesting story, but a rigorous testing of the effects of mutant Httex1 on ribosome stalling is needed to understand the potential impact of this story. Specific concerns and suggestions are listed below.

Major concerns:
1. The authors' claim that "long polyQ sequences do not lead to ribosome stalling" is not currently well-supported. In Figure 1, western blots showed expression of both GFP and mCherry was clearly reduced in the cells transfected with 46Q and 97Q P2A T2A constructs compared to P2A constructs. Are there differences in RNA levels in the cells transfected with these constructs? Additionally, expression of polyQ proteins should be determined by western blot or filter trap assay using anti-Htt or -polyQ antibodies.

10. Please see our response 2 above. The mislabelling had made the data look dramatic for stalling with long polyQ sequences. We believe the manuscript now shows more clearly the arguments for mild stalling at best.

2. CAA interrupted repeats were used for 25Q reporter construct, but no detailed sequences was provided for 46Q and 97Q constructs. Given that both repeat mRNAs and polyQ proteins may cause ribosome stalling, it is necessary to test if ribosomes could stall on CAA interrupted or pure CAG repeats RNAs.

11. We agree that the CAA versus CAG repeat structure would be interesting to assess. However, that is beyond the scope of this current study, which is directed more at the mechanisms of nascent protein-mediated stalling. We have added more detail to the manuscript in Table 1 to include the full nucleotide sequences used for the 25Q, 46Q and 97Q constructs.

3. The authors claim that "the over-expression of these proteins did not appear to influence the formation of inclusions of Httex1(97Q) (Fig 3C)" (lines 264-265). It is not clear however if

Rack1 and Ltn1 have effects on the formation of inclusions. Additionally, the statistical analysis should be performed on impact of HaloTag protein expression on Httex1(97Q)-Cerulean inclusion formation in Fig. 3 and 4.

12. Statistics have been added to the figures and we have revised the results section to more completely explain the findings in context of the statistical analysis.

4. Previous studies showed that degradation and toxicity of polyQ proteins vary in different types of neurons, suggesting ribosome stalling and liquid-like properties of mutant Httex1 may be cell type dependent. These possibilities should be discussed.

13. Without specific studies cited from the reviewer it remains speculative as to how to address this query as there are so many factors that confound comparisons between effects in different cell types.

Minor points

1. In Fig. 1, B and C should be clearly separated. Are western blots of P2A T2A constructs part of Fig.1C?

14. We have revised the figure to clarify these issues.

2. Fig. 1C should be added at the end of the sentence line 197 on page 15.

15. We have made this change.
